# Increasing in-person medical interpreter utilization in the NICU through a bundle of interventions

**DOI:** 10.1038/s41372-024-01915-5

**Published:** 2024-02-29

**Authors:** John Feister, Sheila Razdan, Danielle Sharp, Shamita Punjabi, Elizabeth Blecharczyk, Veronica Escobar, Paw Mar Gay, Melissa Scala, Sonia Bonifacio

**Affiliations:** 1 Stanford University School of Medicine, Department of Pediatrics, Stanford, CA USA; 2 Lucile Packard Children’s Hospital Stanford, Stanford, CA USA; 3 Present Address: University of Cincinnati College of Medicine, Department of Pediatrics & Cincinnati Children’s Hospital Medical Center Division of Neonatology, Cincinnati, OH USA

**Keywords:** Health services, Paediatrics

## Abstract

**Background:**

In-person medical interpretation improves communication with patients who have preferred language other than English (PLOE). Multi-dimensional barriers to use of medical interpreters limit their use in the NICU.

**Local problem:**

Medical teams in our NICU were not consistently using in-person medical interpreters, leading to ineffective communication with families with PLOE.

**Methods/Interventions:**

Interventions included staff educational sessions and grand rounds regarding equitable language access, distribution of interpreter request cards to families, and allocation of dedicated in-person interpreters for NICU rounds. Interpreter utilization was calculated by total requests per Spanish-speaking person day in the NICU.

**Results:**

Interpreter utilization increased five-fold during the intervention period (from 0.2 to 1.0 requests per Spanish-speaking person day).

**Conclusions:**

We substantially increased our unit in-person interpreter utilization through a bundle of multifaceted interventions, many of which were low-cost. NICUs should regard dedicated medical interpreters as a critical part of the care team.

## Introduction

Language barriers affect the care of patients. In the neonatal intensive care unit (NICU), parents with a preferred language other than English (PLOE) report less understanding of their infant’s diagnoses [1, 2] and feel less prepared for discharge [3] than their English-speaking counterparts. Furthermore, infants of Spanish-speaking families are more likely to visit the emergency department [4] and be readmitted to the hospital [5] after NICU discharge.

Limited communication between medical teams and families with PLOE contributes to observed language-based health disparities [6–8]. Medical interpretation can be used to overcome language barriers and improve healthcare delivery [9–12] but availability and ease of use are barriers to interpreter utilization [13–16]. Furthermore, the modality of medical interpretation (i.e., in-person, video, or telephone) can have significant impact on quality of interpretation and user experience [10, 12]. In-person interpretation is generally recognized as the gold standard of medical interpretation [9, 17] but may not be feasible in all settings due to funding constraints or allocation of funds to other resources.

We identified sub-optimal communication between medical teams and families with PLOE as a significant problem in our NICU. Staff reported consistent observations of parents not receiving medical updates in their preferred language, despite available interpreter resources including in-person and video interpretation at our hospital. Qualitative interviews of families with PLOE supported these observations [18]. Furthermore, marked language-based disparities in parental holding of infants, an important developmental care activity, was demonstrated, even when adjusting for rates of parental presence [19]. Appropriate developmental care may provide vital infant and family health benefits including improved physiologic stability, bonding, and neurodevelopmental outcomes [20, 21]. However, vital care processes such as holding may be highly dependent on parental ability to communicate wants and desires with their medical teams, as well as an understanding of the important ways in which parents can contribute to care in the NICU. Given these findings, we created a multi-disciplinary task force focused on providing equitable care to families with a preferred language other than English. Our task force is comprised of NICU physicians, nurse practitioners, social workers, nurses, lactation consultants, patient navigators, and medical interpreters. Our problem statement was “Medical teams are not communicating with families who have PLOE in their preferred language.”

We began systematic tracking of interpreter usage and then designed interventions to increase utilization of in-person interpreters, as this modality is the gold standard of interpretation [9, 17] and available at our institution. We expected that increased use of in-person interpretation would lead to higher quality communication between medical teams and families with PLOE. Our SMART aim was to increase the number of families with a preferred language of Spanish receiving a daily medical update in Spanish by a relative 50% of baseline from January 1, 2022, to January 31, 2023.

## Methods

### Context

Interventions were implemented in a 72-bed quaternary-level NICU within a children’s hospital in Northern California. The patient population includes both premature infants and term infants who require intensive or critical care. Approximately 30% of all patients in this NICU have PLOE, with the vast majority preferring Spanish. Patient preferred language is asked upon admission and documented in the electronic medical record. Our NICU has access to 24/7 in-person Spanish interpretation, as well as video tablet interpretation & telephonic interpretation. In-person interpreters are requested via placing a request in the electronic medical record. Response times for in-person interpreters at our hospital average >20 min during normal morning rounding hours (i.e., “peak hours”) but are much quicker (5–10 min) during off-peak hours.

### Interventions

A multi-disciplinary language access task force of medical interpreters, providers, nurses, social workers, patient navigators, and lactation consultants was formed. A key driver diagram (Supplementary Fig. 1) was created to determine causes of inadequate medical interpreter utilization by staff.

We broadly grouped our interventions into three categories: staff education regarding the need, best practices, and processes for using interpreters; empowering families to request interpretation; and removing barriers to interpreter utilization.

We devised 30-min educational sessions on medical interpretation that were presented at separate meetings with neonatology attendings, neonatology fellows, neonatology advanced practice providers, and NICU nurses. These sessions first emphasized language access standards agreed upon by the language access task force, including who should use interpreters, when interpreters should be used, and what modality of interpretation was appropriate for which situations (Supplementary Fig. 2). We then demonstrated the step-by-step process for requesting a medical interpreter via our electronic medical record. We also presented a NICU Health Equity Grand Rounds related to language access that included actionable changes providers could make to promote language equity in their practice.

We simultaneously implemented a modification to our daily NICU census and safety huddle (attended by on-service providers and nursing staff) to identify families in the NICU with a PLOE and remind staff to use a medical interpreter when communicating medical information. We also incorporated preferred language into the patient identifier section of our provider handoff tool and in daily progress notes.

To empower families to request an interpreter, we created a bilingual interpreter request card (Supplementary Fig. 3) that they could hand to staff. These cards were distributed on admission by our bilingual patient navigator. Our medical interpreters also reminded families of their right to medical interpretation whenever they communicated with families.

Our final interventions focused on removing barriers to utilizing medical interpreters. We were able to secure an in-person Spanish medical interpreter for 2 h each weekday who was exclusively stationed in the NICU (in addition to the previously available hospital-wide in-person interpreters). This interpreter was already a member of the hospital medical interpreter staff, but prior to this intervention had not been designated as a “NICU-specific” interpreter. The interpreter accompanied medical teams during rounds and was available immediately after for family conferences and education sessions. To maximize efficiency and flexibility of interpretation, a daily group chat on a secure electronic messaging system amongst providers and the interpreter was created so that the interpreter could “float” between the three NICU teams when needed.

We also developed a pilot program of nighttime family-centered interpreter rounds, where the overnight medical team gave updates via an already available hospital-wide in-person interpreter to families who were only able to be present at night. This intervention was facilitated by creating a process in which the hospital interpreter, if not busy with other patient needs, came to the NICU on a nightly basis and checked in with the medical team to see if there were any needs for interpretation.

### Study of interventions

We assessed the impact of our interventions over time by determining the number of in-person and video tablet requests per Spanish-speaking person day in the NICU. At our institution, in-person interpreter requests are placed through the electronic medical record. Tablet and phone interpretation encounters are tracked by unit in which they were used but have no linkage to the electronic medical record.

The number of in-person interpreter requests and video tablet interpreter uses in the NICU per month were collected via the electronic medical record system and the video tablet company (AMN Healthcare; Coppell, TX). The number of patient days (i.e., 7-day stay of 1 patient = 7 patient days) of families with Spanish or a Central American indigenous language (i.e., Mixteco, Triqui, Zapoteco, Mam) in the NICU for each month were calculated. We chose to include patients with Central American indigenous languages in the patient day calculation since in-person Spanish interpreters are commonly used at our institution to facilitate interpretation of indigenous languages via third-party telephonic interpreters.

We then divided the total number of in-person and video tablet interpreter uses by the total number of patient days to get a rate of interpreter utilization.

This work was deemed quality improvement and thus no IRB was submitted. This manuscript was developed using guidelines from the Revised Standards for Quality Improvement Reporting Excellence (SQUIRE 2.0).

## Results

**Fig. 1:**
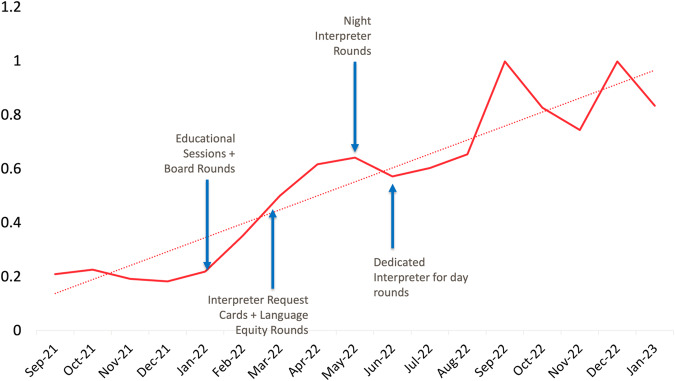
Number of in-person Spanish interpreter requests per Spanish-speaking patient day, by month.

**Fig. 2:**
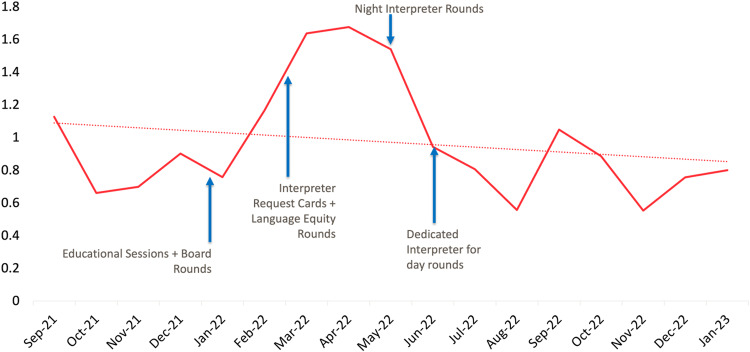
Number of video tablet Spanish interpreter sessions per Spanish-speaking person day, by month.

**Fig. 3:**
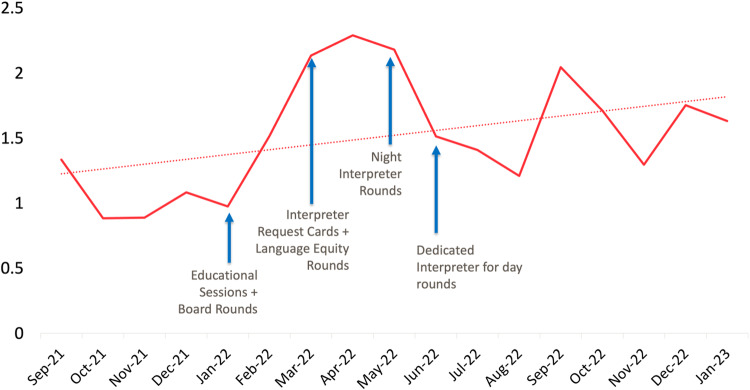
Total number of in-person + video tablet Spanish interpreter requests per Spanish-speaking person day, by month.

In the four months preceding implementation of our interventions (September–December 2021), the in-person interpreter request per Spanish-speaking person day rate was 0.2. There was an immediate increase in in-person interpreter requests in January 2022 concurrent with initiation of educational sessions, and the rate steadily increased throughout our intervention period to 1.0 requests per Spanish-speaking person day in December 2022 (Fig. 1). There was a noticeable increase in in-person interpreter utilization after starting daily interpreter rounds in June 2022. There was an initial increase in video tablet usage that stabilized to near baseline levels by the end of the study period (Fig. 2). There was an overall increase in the aggregate usage of in-person & video tablet interpreters during the study period (Fig. 3).

## Discussion

Through a bundle of multifaceted interventions, we were able to increase our NICU’s utilization of in-person Spanish interpreters five-fold above the baseline while simultaneously increasing overall usage of all modes of medical interpretation. Importantly, several of our interventions took little effort & cost to implement and had immediate effects.

Significant improvements in in-person interpreter utilization were seen with each intervention we implemented. Staff educational sessions & health equity grand rounds focused on language access were associated with a three-fold increase in in-person interpreter utilization. These interventions had no associated cost beyond time and effort. These educational interventions primed our unit to maximize the impact of a dedicated in-person interpreter once their services were secured for daily patient rounds.

Removing barriers to use of high-quality medical interpretation is a powerful tool to ensure that families receive communication from the medical team in their preferred language [13, 16]. Installation of a dedicated unit interpreter led to a significant increase in interpreter utilization. Additionally, having a dedicated in-person interpreter had a profound impact on unit culture regarding language access. The interpreter was perceived as part of the team, rather than a cumbersome service. This intervention created a feedback loop, where, as the team realized the ease of use, built trust with, and experienced superior communication with families through the in-person interpreter, there was a natural desire to continue working with the interpreter. This feedback loop steadily increased interpreter utilization. We believe this slow but steady culture change regarding language access is the most impactful aspect of our work.

Our work aligns with prior limited literature investigating methods to increase interpreter utilization. A similar multifaceted quality improvement project at a large children’s hospital in the Pacific Northwest increased telephonic interpreter utilization rates from 0.38 uses per person day to 0.58 [15]. A health system-wide initiative focused on improving language access in Michigan for adult patients with diabetes resulted in an increase from 19% to 83% in the proportion of outpatient visits for patients with PLOE with a qualified language services provider present [22]. Our work demonstrates a similarly low initial level of interpreter usage with a marked increase following focused interventions. Concordant with our work, both cited studies relied heavily on staff education regarding the importance of medical interpretation and removal of barriers to existing language access resources.

Our efforts have a few distinguishing features from prior work. First, we concentrated on increasing in-person interpretation (rather than telephonic interpretation), as this modality is widely considered the gold standard of medical interpretation and our hospital had existing resources in place to allow for frequent use of in-person interpreters. Second, our work was in an ICU setting, where complex medical discussions with families routinely occur and optimal communication is of utmost importance. Last, we made a conscious effort to educate families on their right to medical interpretation and empower them to request interpreters.

It is critical to note that our interventions led to an overall increase in use of any interpreter modality (i.e., both in-person and video). Thus, we did not find evidence of staff limiting interactions with families when it was not feasible to use an in-person interpreter. Rather, the in-person interpreter was preferentially used, but video interpretation remained widely employed. We hypothesize that the initial surge in video interpretation in the beginning of our intervention period was related to increased awareness of need for medical interpretation but limited ability to access in-person interpretation. Our video interpretation utilization returned to baseline levels as we removed barriers to in-person interpretation (i.e., implemented a dedicated NICU medical interpreter). This pattern of interpreter modality use was consistent with our suggested language access guidelines presented during staff educational sessions.

We encountered several challenges during our work that may be informative for other hospitals focusing on improving language access. First, we had initial difficulty understanding our pattern of interpreter utilization at baseline, as there was no dedicated process in place to track interpreter use. We were able to better study our interpreter use after creation of monthly reports of total in-person and tablet requests by using available data from the electronic medical record and video tablet interpretation company. Second, cost and staffing were two major constraints that initially prevented us from obtaining a dedicated in-person interpreter for the NICU. A dedicated NICU interpreter was approved on a pilot basis; given her impact and overwhelmingly positive feedback from NICU staff and families, members of our task force were successful in obtaining grant funding for a full-time NICU interpreter.

There are several limitations to our work. First, we did not track interpreter requests for individual patients. For example, our current data shows only the total number of interpreter requests for any given day – not if each individual family received an update via a medical interpreter. Future work may focus on understanding patterns of interpreter utilization for individual patients (rather than aggregate interpreter utilization in the NICU). Second, our work did not delineate what type of communication occurred when the in-person interpreter was requested. For example, we could not ascertain from our data whether the interpreter request was for a direct medical update from a provider or for another reason (i.e., orientation to the unit, equipment education, etc.). Lastly, many of our neonatal health outcomes are not systematically tracked by language, limiting our ability to describe associations between our work and other health outcomes such as receipt of human milk, length of stay, & readmission after discharge, among others.

In conclusion, we substantially increased our unit in-person interpreter utilization through a series of multifaceted interventions, many of which were low-cost. The ability to communicate with the medical team is a patient right and an important driver of health equity. We encourage NICUs to systematically study patterns of interpreter utilization to identify areas of improvement. Finally, we advocate for NICUs to view an in-person interpreter as a dedicated and integral part of the NICU team.

## Supplementary information

Supplemental Figures 1,2,3
