# Supplementary material for: Increasing in-person medical interpreter utilization in the NICU through a bundle of interventions
Source: J Perinatol. 2024 Feb 29;45(2):273–7. doi: 10.1038/s41372-024-01915-5 (PMC11825357; doi:10.1038/s41372-024-01915-5)
Supplement: Supplementary file 1 — Supplemental Figures 1,2,3 [file 41372_2024_1915_MOESM1_ESM.docx]

Supplementary figures


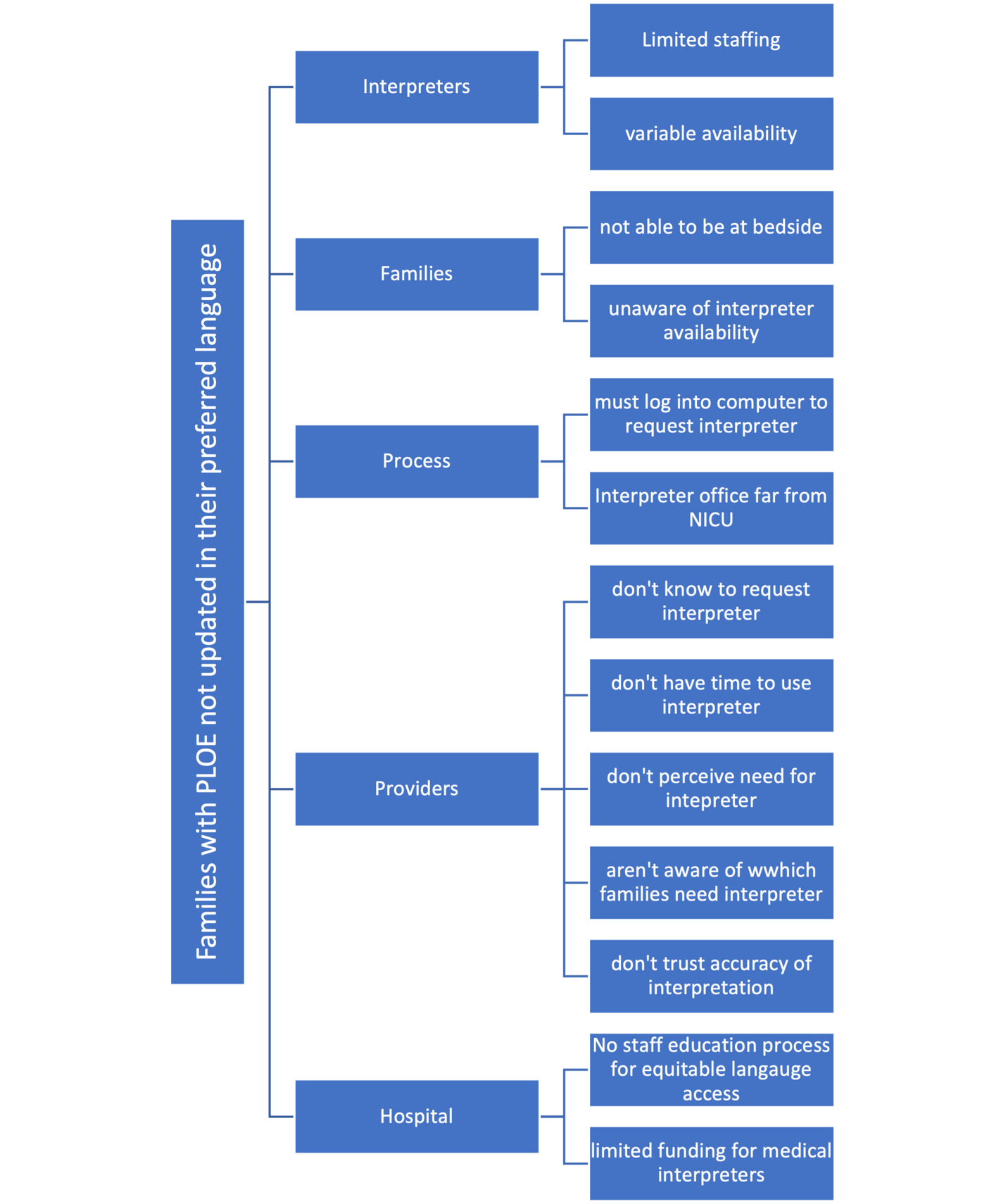


*Supplemental Figure 1. Fishbone diagram describing potential causes of families not receiving updates in their preferred language.*


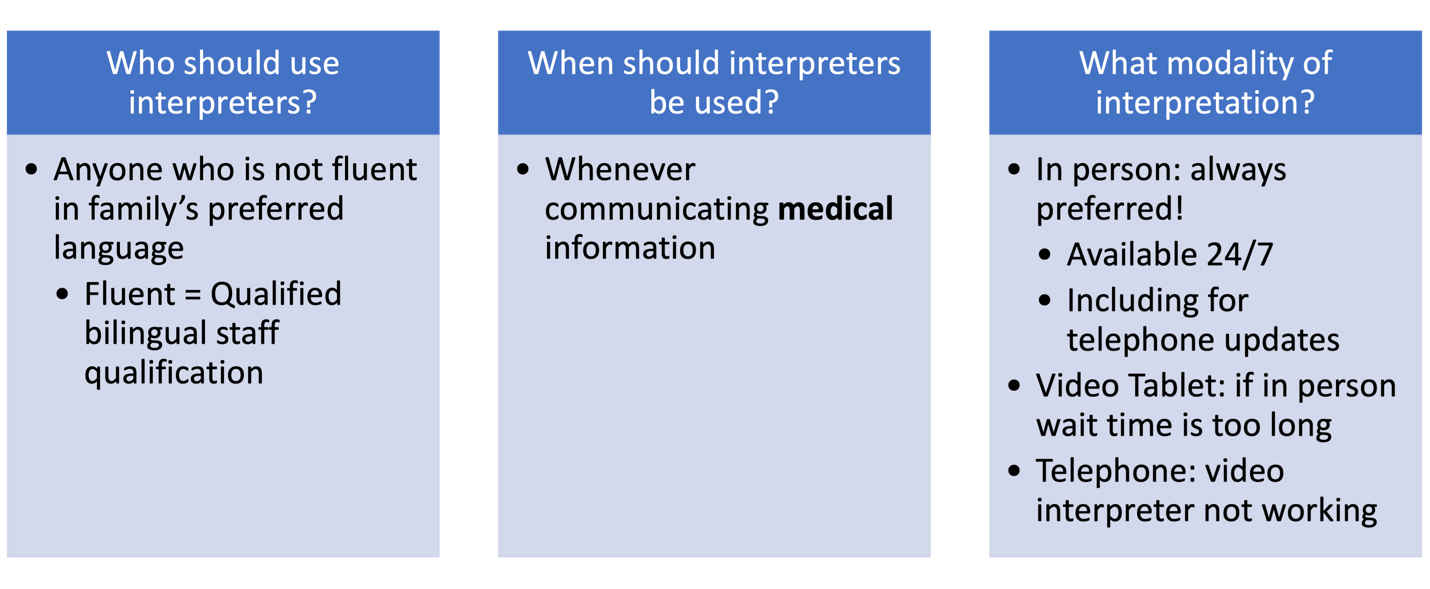


*Supplemental Figure 2. Interpreter use guidelines presented at staff educational sessions.*


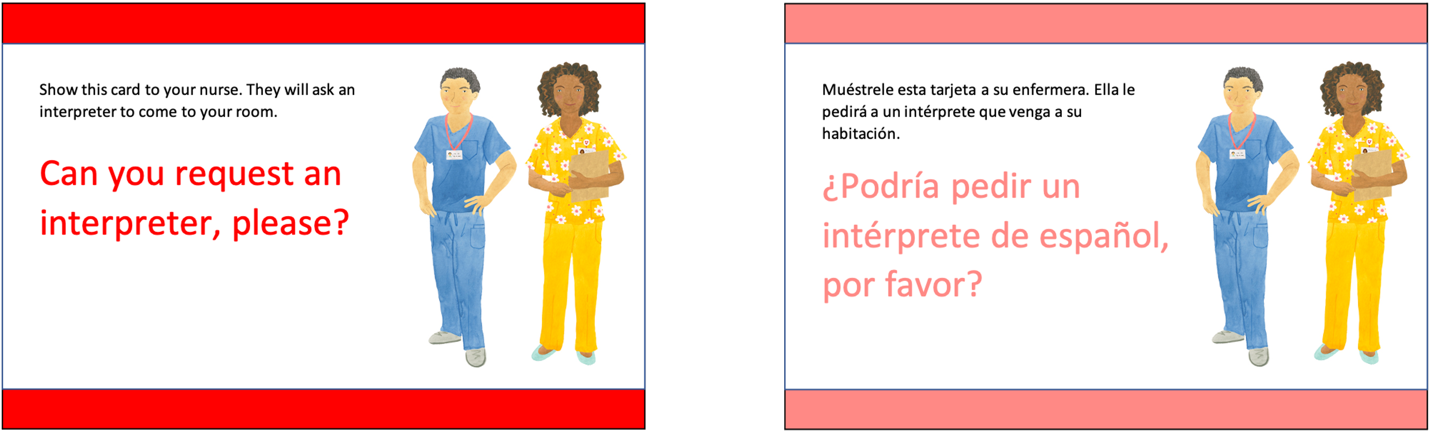


*Supplemental Figure 3. Interpreter request cards (double sided) distributed to Spanish speaking families.*
